# Role of vertebral corner inflammation and fat deposition on MRI on syndesmophyte development detected on whole spine low-dose CT scan in radiographic axial spondyloarthritis

**DOI:** 10.1136/rmdopen-2022-002250

**Published:** 2022-07-08

**Authors:** Rosalinde Stal, Xenofon Baraliakos, Désirée van der Heijde, Floris van Gaalen, Sofia Ramiro, Rosaline van den Berg, Monique Reijnierse, Juergen Braun, Robert Landewé, Alexandre Sepriano

**Affiliations:** 1 Rheumatology, Leiden University Medical Center, Leiden, The Netherlands; 2 Rheumazentrum Ruhrgebiet, Ruhr University Bochum, Bochum, Germany; 3 Rheumatology, Zuyderland Medical Centre Heerlen, Heerlen, The Netherlands; 4 Radiology, Leiden University Medical Center, Leiden, The Netherlands; 5 Rheumatology, Amsterdam UMC Locatie AMC, Amsterdam, The Netherlands; 6 Rheumatology, NOVA Medical School, Universidade Nova de Lisboa, Lisbon, Portugal

**Keywords:** inflammation, magnetic resonance imaging, spondylitis, ankylosing

## Abstract

**Objectives:**

To investigate the associations between MRI detected vertebral corner inflammation (VCI) and vertebral corner fat deposition (VCFD) on whole spine low-dose CT scan (ldCT) detected syndesmophyte formation and growth.

**Methods:**

Patients from the Sensitive Imaging in Ankylosing Spondylitis cohort underwent MRI (baseline, 1 year and 2 years) and ldCT (baseline and 2 years). MR images were scored by three readers for VCI and VCFD, MRI patterns were defined by presence of VCI and/or VCFD over 2 years. LdCT images were scored by two central readers for presence and size of syndesmophytes and change was calculated for new or new/grown syndesmophytes. Multilevel generalised estimated equations were used to test the associations between VCI and VCFD and syndesmophyte development.

**Results:**

Fifty radiographic patients with axial spondyloarthritis were included (mean age 49 years, 86% male, 78% HLA-B27+). Absence of both VCI and VCFD protected against syndesmophyte development (ORs 0.36–0.37). Presence of VCI and/or VCFD increased the risk of syndesmophyte development (ORs 1.73–2.60). Out of all corners with a new or new/grown syndesmophyte, 47% of corners according to reader 1 and 44% according to reader 2 had neither VCI nor VCFD preceding the bone formation.

**Conclusions:**

VCI and VCFD were positively associated with syndesmophyte development. This has been shown for the first time for syndesmophytes detected on ldCT and also in the thoracic spine. However, almost half of all bone formation occurred in corners without VCI or VCFD, suggesting the presence of these lesions in yearly MRIs does not fully clarify the development of syndesmophytes.

**WHAT IS ALREADY KNOWN ON THIS TOPIC**

- Associations between vertebral corner inflammatory and fatty lesions and syndesmophyte development have been reported in anterior corners of the cervical and lumbar spine on conventional radiography (CR).

**WHAT THIS STUDY ADDS**

- Vertebral corner inflammation and fat are, both together and separately, associated with syndesmophyte formation when studying all spinal levels and both anterior and posterior corners and using low-dose CT scan (ldCT) for syndesmophyte detection.
- Inflammation and fat have similar effects on the formation and growth of syndesmophytes.
- The finding of these associations on ldCT and in the whole spine confirms the existence and magnitude of these associations.

**HOW THIS STUDY MIGHT AFFECT RESEARCH, PRACTICE AND/OR POLICY**

- LdCT can be used to study and monitor the reported effects in the whole spine, thus trials can use the benefits of ldCT over CR in terms of increased visibility.

## Introduction

Radiographic axial spondyloarthritis (r-axSpA) is a chronic inflammatory disease causing pain and movement limitation.^1^ In r-axSpA, bone proliferation can occur in the sacroiliac joints and, in a subset of patients, the spine, which can lead to severe spinal deformities. Understanding the pathways leading to bone proliferation is crucial in identifying effective treatment strategies to prevent spinal damage.

Several studies have been performed on the associations between vertebral corner inflammation (VCI) and vertebral corner fat deposition (VCFD) on spinal MRI and syndesmophyte development on conventional radiography (CR).^2–7^ All studies reported an association between the MRI lesions and syndesmophyte development, although the strengths of the associations differed somewhat across studies. Furthermore, all studies reported syndesmophytes to also develop in vertebral corners without VCI or VCFD.

Since the previous studies used CR for syndesmophyte detection, which assesses syndesmophytes in the cervical and lumbar spine and on anterior vertebral corners only, the association between MRI lesions and syndesmophyte development has not yet been studied in the thoracic spine, nor in the posterior corners. Low-dose CT scan (ldCT) enables the assessment of the thoracic spine, as well as the assessment of syndesmophyte growth in addition to new syndesmophyte formation. In the current study, we assessed the effects of VCI and VCFD on the development of ldCT detected syndesmophyte formation and growth in the whole spine.

## Methods

### Study population

Data were used from the Sensitive Imaging in Ankylosing Spondylitis (SIAS) cohort, which included patients from the Netherlands and Germany with a clinical diagnosis of r-axSpA, who also fulfilled the modified New York criteria. Patients had ≥1 and ≤18 syndesmophytes on CR of the lateral cervical and lumbar spine assessed with the modified Stoke Ankylosing Spondylitis Spinal Score and ≥1 inflammatory lesion on spinal MRI.

### Imaging

Patients underwent whole spine ldCT at baseline and 2 years and whole spine MRI at baseline, 1 year and 2 years.

LdCT images were obtained on a 64-section and 16-section CT scanner (Leiden: Aquilion 64, Toshiba Medical Systems, Otawara, Japan; Herne: Somatom Emotion 16, Siemens, Erlangen, Germany). Spiral CT scan was performed with 60 mAs at 120 kVp and a pitch of 53/64 using automatic exposure control with 30 SD/60 reference mAs. The images were scored independently by two trained central readers on sagittal and coronal images on 2 mm slices using the CT Syndesmophyte Score (CTSS).^8^ In short, the CTSS assesses four vertebral quadrants per vertebral unit (VU) per plane on a 4-point scale: 0, no syndesmophyte; 1, syndesmophyte reaching<50% of the intervertebral disc space (IDS); 2, syndesmophyte reaching≥50% of the IDS; and 3, bridging syndesmophyte. A VU is defined as the bottom half of a vertebra, the upper half of the vertebra underneath and the IDS in between.

MR images were acquired in Leiden and Herne, respectively, on a 3T MRI (Philips Medical systems, Best, The Netherlands) and 1.5T MRI (Siemens, Erlangen, Germany) using sagittal T1-weighted and short tau inversion recovery (STIR) sequences with a slice thickness of 3 mm. Detailed information about both scanning techniques is described elsewhere.^8^ Three trained central readers scored VCI on STIR images according to the Spondyloarthritis Research Consortium of Canada (SPARCC) method and VCFD on T1-weighted images according to the CanDen method.^9 10^

MRI and ldCT images were scored per modality, with the readers blinded for time order, clinical information and the results of the other modality. The current study uses only ldCT scores from the sagittal plane to match the plane in which MRI images were scored.

### Variable definitions

Patterns of MRI lesions are made on the vertebral corner level and describe hypothetical associations between VCI and VCFD and the development of syndesmophytes on the same corner. Patterns are based on patterns studied by Machado *et al*.^6^ Three categories of patterns are studied: patterns regarding VCI only, regarding VCFD only and regarding both (online supplemental table 1). At each of the three timepoints (baseline, 1 year and 2 years), the presence of VCI and VCFD is coded binary per reader. Then, it is determined per reader whether a pattern of lesions over time is present. For example, the pattern of VCI at any timepoint (pattern 1, online supplemental table 1) is present for a reader when there is presence of VCI on at least one of the three timepoints, irrespective of presence of VCFD. Finally, a binary consensus score is made if the pattern was observed by ≥2 out of 3 MRI readers. The models use only the binary consensus scores as predictors.

10.1136/rmdopen-2022-002250.supp1 Supplementary data

Syndesmophyte development was calculated in two ways: as the formation of a new syndesmophyte and as either the formation of a new syndesmophyte or the growth of an existing syndesmophyte. Preferably, two out of the three agreement scores would also be made for the ldCT scores. However, ldCT images were only scored by two readers and the choice was made to use individual reader ldCT scores in the models. For each of the two ldCT readers, binary change scores were made reflecting the new (change in CTSS from 0 to 1, 2 or 3) and new/grown (change in CTSS from 0 to 1, 2 or 3; from 1 to 2 or 3; or from 2 to 3) syndesmophytes. In contract to the MRI scores, ldCT scores were not combined into consensus scores but rather individual reader change scores were used. Corners not at risk for the outcome due to presence of a syndesmophyte at baseline for the new syndesmophyte change score (CTSS 1, 2 or 3) and presence of a bridged syndesmophyte at baseline for the new/grown syndesmophyte change score (CTSS 3) were excluded. In addition, corners could have a missing CTSS score because the reader deemed the corner could not be assessed due to image quality. Missing vertebral corner scores were not imputed and resulted in a missing individual reader change score.

### Statistical analysis

Models were made on the vertebral corners level per MRI pattern and syndesmophyte outcome (either new or new/grown). Generalised estimated equation models were used for all analyses to account for the within-patient and within-ldCT reader correlations. The within-patient correlation was taken into account by adjusting for the corner level, by specifying in the model which vertebral corners belong to the same patient using the exchangeable correlation structure. In the same way, a variable was added indicating which ldCT reader scores belonged to the same corner. The variable specifying the reader level only adjusts for the ldCT reader scores, since agreement scores were used for MRI patterns. Based on prior studies on risk factors for syndesmophyte development, each model was adjusted for age, sex and smoking status (ever/never) at baseline.^11^ All analyses were performed in Stata V.16.

## Results

In total, 50 patients underwent ldCT and MRI at all scheduled timepoints and were included in the analyses (mean age 49.3 years (SD 9.8), 86% male, 78% HLA-B27+)(online supplemental table 2).

Per patient, four vertebral corners of 23 VUs were scored, giving a total of 4600 vertebral corners per timepoint. After excluding corners not at risk and corners with missing CTSS scores, 2880 and 2811 corners were analysed for the development of new syndesmophytes for readers 1 and 2, respectively, and 3251 and 3232 corners were analysed for development of new/grown syndesmophytes for readers 1 and 2, respectively.

The mean effective dose estimates were 4.3 mSv (SD 2.5 mSv) for all ldCT scans made in the SIAS cohort (Dose Length Product: mean 304 mGy×cm (SD 181 mGy×cm); CT Dose Index_vol_: mean 5.1 mGy (SD 2.7 mGy). The effective dose is currently estimated to be as low as 1.4–1.7 mSv per ldCT using technical optimisation in the 64-slice scanners, without noticeable imaging quality loss.

### Presence of syndesmophytes and MRI patterns

Percentages of missing syndesmophyte scores were low overall, but slightly more frequent in the cervicothoracic area. New syndesmophytes were reported in 144 out of 2880 corners (5%) for reader 1. When analysed per segment, new syndesmophytes occurred in 5% of corners in the cervical spine (41/792), 6% in the thoracic spine (82/1408) and 3% in the lumbar spine (21/680). New syndesmophytes were reported in 185 out of 2811 corners (7%) for reader 2. Per segment, new syndesmophytes occurred in 4% of corners in the cervical spine (29/714), 10% in the thoracic spine (136/1403) and 3% in the lumbar spine (20/694). New or grown syndesmophytes were reported in 205 out of 3251 corners (6%) for reader 1. When analysed per segment, new or grown syndesmophytes occurred in 6% of corners in the cervical spine (57/872), 7% in the thoracic spine (110/1567) and 5% in the lumbar spine (38/812). New or grown syndesmophytes were reported in 264 out of 3232 corners (8%) for reader 2. Per segment, new or grown syndesmophytes occurred in 6% of corners in the cervical spine (48/800), 11% in the thoracic spine (180/1599) and 4% in the lumbar spine (36/833). Thus, for reader 1, the rates of developed (new or new/grown) syndesmophytes were slightly higher in the cervical and thoracic spine compared with the lumbar spine, and for reader 2, most progression was reported in the thoracic spine.

**Figure 1:**
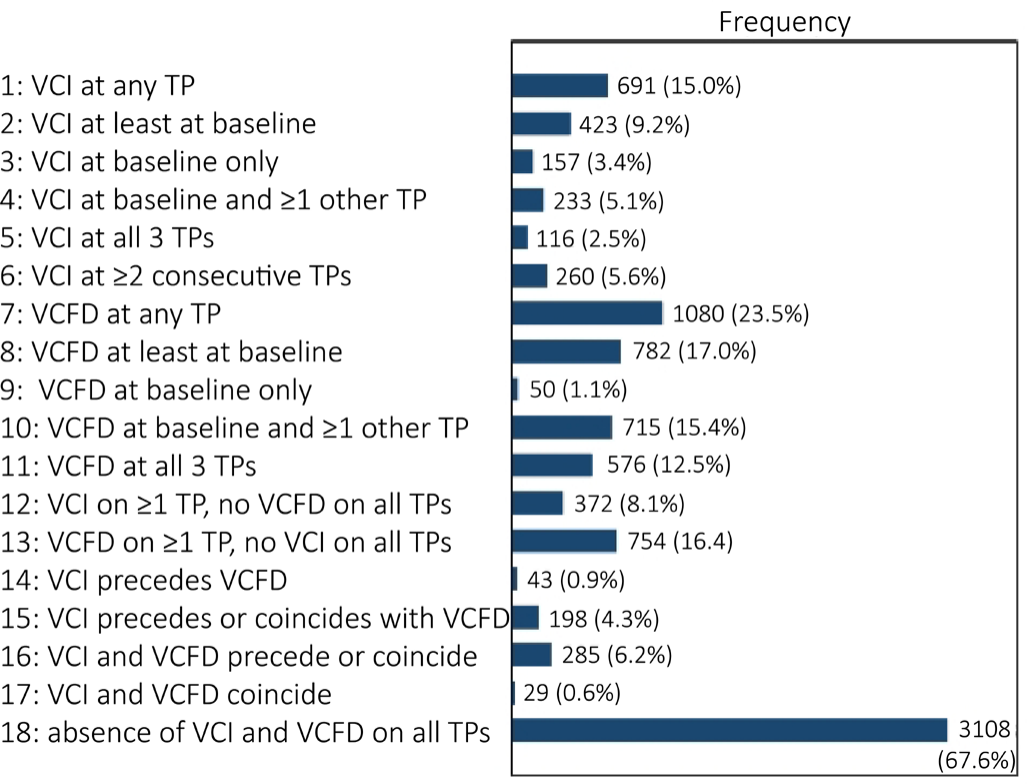
Observed frequencies of patterns of MRI lesions. Patterns over time were studied in a total of 4600 vertebral corners. Patterns are deemed present if ≥2 out of 3 MRI readers scored the pattern over time. The patterns are not all mutually exclusive. For example, of the 4600 corners, 116 had presence of VCI at all timepoints (pattern 5). These corners are all included in the 691 corners with presence of VCI on at least one timepoint (pattern 1), and presence of VCFD is disregarded in these patterns. TP, timepoint; VCFD, vertebral corner fat deposition; VCI, vertebral corner inflammation.

The frequency of observed MRI patterns ranged from 29 (1%) to 3108 (68%) out of all 4600 corners (figure 1).

### Association between MRI patterns and syndesmophytes

VCI and VCFD were both positively associated with the development of new and new/grown syndesmophytes for various patterns when studied regardless of the other (table 1, patterns 1–11). For VCI, significant positive ORs were found for 4/6 patterns for associations with new syndesmophytes (range ORs 2.20–2.53) and for 5/6 patterns for associations with new/grown syndesmophytes (range ORs 2.14–2.60). VCI at baseline only (pattern 3) was present in 157 out of 4600 corners (3.4%) and had an OR in the same range (2.35) but was the only pattern not significantly associated with syndesmophyte development. For VCFD, 4/5 patterns were associated with new (range ORs 2.28–2.58) and new/grown (range ORs 2.29–2.44) syndesmophytes. VCFD at baseline only (pattern 9) was present in 50 out of 4600 corners (1%) and was not significantly associated with syndesmophyte development (OR 0.63).

**Table 1:** Effect of vertebral corner inflammation (VCI) and vertebral corner fat deposition (VCFD) on syndesmophyte development and growth

Patterns of lesions over time on MRI	New syndesmophyte OR (95% CI)	New/grown syndesmophyte OR (95% CI)
Regarding VCI only		
VCI at any TP	**2.20 (1.23 to 3.93**)	**2.20 (1.37 to 3.52**)
VCI at least at baseline, irrespective of VCI at other TPs	**2.53 (1.32 to 4.84**)	**2.60 (1.63 to 4.15**)
VCI at baseline only	2.47 (0.78 to 7.77)	2.35 (1.00 to 5.54)
VCI at baseline and at least one other TP	**2.50 (1.41 to 4.44**)	**2.42 (1.56 to 3.74**)
VCI at all three TPs	1.93 (0.87 to 4.29)	**2.16 (1.25 to 3.72**)
VCI at ≥2 consecutive TPs	**2.25 (1.14 to 4.41**)	**2.14 (1.22 to 3.77**)
Regarding VCFD only		
VCFD at any TP	**2.30 (1.69 to 3.12**)	**2.42 (1.86 to 3.16**)
VCFD at least at baseline, irrespective of VCFD at other TPs	**2.28 (1.68 to 3.09**)	**2.29 (1.78 to 2.95**)
VCFD at baseline only	*	0.63 (0.19 to 2.07)
VCFD at baseline and at least one other TP	**2.47 (1.83 to 3.33**)	**2.44 (1.91 to 3.11**)
VCFD at all three TPs	**2.58 (1.80 to 3.69**)	**2.33 (1.70 to 3.18**)
Regarding both VCI and VCFD		
VCI on ≥1 TP and absence of VCFD on all TPs	**2.26 (1.32 to 3.89**)	**1.87 (1.11 to 3.13**)
VCFD on ≥1 TP and absence of VCI on all TPs	**2.15 (1.56 to 2.98**)	**1.91 (1.43 to 2.54**
VCI precedes VCFD	1.84 (0.51 to 6.74)	2.51 (0.88 to 7.17)
VCI precedes or coincides with VCFD. VCFD does not precede VCI	1.84 (0.93 to 3.64)	**2.37 (1.40 to 4.00**)
VCI and VCFD precede each other or coincide	1.73 (0.82 to 3.63)	**2.12 (1.18 to 3.82**)
VCI and VCFD coincide at the same TP	2.04 (0.42 to 9.76)	2.61 (0.90 to 7.57)
Absence of VCI and VCFD on all TPs	**0.36 (0.26 to 0.51**)	**0.37 (0.27 to 0.52**)

Each OR is derived from a separate multilevel GEE model, in which the effect of the MRI pattern and the outcome is assessed at the vertebral corner level. MRI scores are used as consensus scores, and ldCT scores are used as individual reader scores. Each model is adjusted for within-patient and within-ldCT reader correlations, as well as age, sex and smoking status never/ever. Statistical significance is indicated in bold. * predictor is omitted from the model for predicting failure perfectly.

GEE, generalised estimating equations; ldCT, low-dose CT; TP, timepoint.

The effects of VCI and VCFD persisted when studied in absence of the other (patterns 12, 13), while patterns assessing the order of presence of VCI and VCFD occurred between 0.6% and 6.2% and yielded overall non-significant associations (patterns 14–17). Absence of both VCI and VCFD on all timepoints (pattern 18) occurred most frequently (3108/4600 corners (67.6%)) (figure 1) and protected against new (OR 0.36) and new/grown (OR 0.37) syndesmophytes. All models were adjusted for age, sex and smoking based on literature, none of these factors had significant impact on the results.

A significant portion of syndesmophytes developed in corners without any VCI or VCFD present on any of the timepoints. For ldCT reader 1, 74 out of the 144 corners (51%) that developed a new syndesmophyte and 97 out of the 205 corners (47%) that developed a new or a grown syndesmophyte had absence of VCI and VCFD on all timepoints (defined by the MRI pattern, ie, ≥2 out of 3 MRI readers agree on absence of VCI and VCFD on all three timepoints). For ldCT reader 2, 83 out of the 185 corners (45%) with a new syndesmophyte and 116 out of the 264 corners (44%) that developed a new or a grown syndesmophyte had absence of VCI and VCFD on all timepoints.

## Discussion

In this study, we found that presence of both VCI and VCFD, in yearly MRIs, increases the odds of the formation and/or growth of syndesmophytes in the same vertebral corner within 2 years in patients with r-axSpA with at least one pre-existing syndesmophyte. The study was performed in an observational cohort, allowing us to study these associations in a setting representing daily clinical practice and all models were adjusted for age, sex and smoking. This study adds to the existing literature by showing that inflammation and fat not only drive syndesmophyte formation but also its growth. One major advantage of our study compared with previous studies is the use of ldCT. To our knowledge, this is the first study assessing the association between VCI, VCFD and syndesmophytes in whole spine, compared with only in the cervical and lumbar anterior corners as was done in previous studies.^5–7^ This allowed us to demonstrate that the effects under study are also seen when studying the whole spine including the thoracic spine and the posterior vertebral corners. The thoracic spine is of specific interest since it has been shown that most damage occurs here.^12^ Assessing the whole spine also allowed us to have a large sample of vertebral corners when having a modest sample size of 50 patients.

No specific pattern of VCI or VCFD over time was found to contribute most to syndesmophyte development. Of note, the presence of VCI and VCFD at baseline only was uncommon in our population as was the sequence in which VCI precedes the development of VCFD (0.9%) or coincides with VCFD (0.6%). Low occurrence of these patterns might explain, at least partially, their lack of association with the development of syndesmophytes, contrary to what was found in the previous study by Machado *et al*.^6^ Whether or not VCFD mediates the effect of VCI on new bone formation needs further evaluation.

A protective effect was found for the absence of VCI and VCFD at all timepoints on syndesmophyte development. However, about half (45%–51%) of new syndesmophytes developed in corners without VCI or VCFD at all timepoints. This is generally in line with the percentages reported in other studies. In those studies, 46%–57% of the corners with new syndesmophytes had absence of VCI and VCFD at baseline,^5 7^ 40%–66% had absence of VCI and VCFD at all three timepoints^6^ and 62%–76% had absence of VCI at baseline (no VCFD assessed).^2–4^ The development of new syndesmophytes at 2 years was 1.5–2.5 times as high for our own study (5%–7%) compared with these previous studies (2%–5%), which probably reflects the inclusion of the thoracic spine and the use of ldCT.

Several factors could explain why syndesmophyte development is found in absence of VCI and VCFD. One possible explanation is that the methods used, such as number of timepoints, the length of time intervals and the imaging methods for the predictor and outcome, could not fully capture VCI, VCFD and syndesmophyte development. However, by finding similar results as previous studies while using whole spine ldCT instead of cervical and lumbar CR, our study shows that the phenomenon is not likely caused by the method used for syndesmophyte detection or the segments of the spine under study. Possibly the use of more and more frequent MRI examinations could partially reduce the effect by increasing VCI and VCFD detection. Another explanation for the consistent finding that VCI and VCFD do not fully explain syndesmophyte development is that there are other pathophysiological mechanisms at play.

Regarding our statistical methods, it should be noted that our models were not adjusted for treatment. This is a factor that has been adjusted for in some previous studies,^4 6^ but based on causal reasoning it was decided against in the current study. Medication use and inflammation on the vertebral corner level likely affect each other in the sense that presence of preceding VCI can be an incentive to start or switch medication, which, in turn, can subsequently lower presence of VCI. While VCI is associated with syndesmophyte development, medication use is, to our current knowledge, not directly associated with the syndesmophyte development but rather through its effect of reducing inflammation.^13^ Hence, adjusting for medication when studying the association between VCI and syndesmophyte development could erroneously remove part of the total effect.

In summary, this study showed that presence of VCI and VCFD in yearly MRIs increases the odds of formation and growth of syndesmophytes on whole spine ldCT in the same vertebral corner 2 years later in patients with r-axSpA.

## Data Availability

The data underlying this article will be shared on reasonable request to RS.
