# Supplementary material for: Role of vertebral corner inflammation and fat deposition on MRI on syndesmophyte development detected on whole spine low-dose CT scan in radiographic axial spondyloarthritis
Source: RMD Open. 2022 Jul 8;8(2):e002250. doi: 10.1136/rmdopen-2022-002250 (PMC9272129; doi:10.1136/rmdopen-2022-002250)
Supplement: Supplementary data [file rmdopen-2022-002250supp001.pdf]

Supplementary tables

Supplementary table 1: Detailed definitions of patterns of MRI lesions.

**Regarding VCI only**	**baseline**	**1-year**	**2-years**
1--VCI at any timepoint, irrespective of VCFD status	**VCI+**	VCI+/-	VCI+/-
	VCI+/-	**VCI+**	VCI+/-
	VCI+/-	VCI+/-	**VCI+**
2--VCI at baseline, irrespective of VCI status at other timepoints and irrespective of VCFD status	**VCI+**	VCI+/-	VCI+/-
3--VCI at baseline only, irrespective of VCFD status	**VCI+**	**VCI-**	**VCI-**
4--VCI at baseline and another timepoint, irrespective of VCFD status	**VCI+**	**VCI+**	VCI+/-
	**VCI+**	VCI+/-	**VCI+**
5--VCI at all three timepoints, irrespective of VCFD status	**VCI+**	**VCI+**	**VCI+**
6--VCI at ≥ 2 consecutive timepoints, irrespective of VCFD status	**VCI+**	**VCI+**	VCI+/-
	VCI+/-	**VCI+**	**VCI+**
**Regarding VCFD only**	**T0<br>baseline**	**T1<br>1-year**	**T2<br>2-years**
7--VCFD at any timepoint, irrespective of VCI status	**VCFD+**	VCFD+/-	VCFD+/-
	VCFD+/-	**VCFD+**	VCFD+/-
	VCFD+/-	VCFD+/-	**VCFD+**
8--VCFD at baseline, irrespective of VCFD status at other timepoints and irrespective of VCI status	**VCFD+**	VCFD+/-	VCFD+/-
9--VCFD at baseline only, irrespective of VCI status	**VCFD+**	**VCFD-**	**VCFD-**
10--VCFD at baseline and another timepoint, irrespective of VCI status	**VCFD+**	**VCFD+**	VCFD+/-
	**VCFD+**	VCFD+/-	**VCFD+**
11--VCFD at all three timepoints, irrespective of VCI status	**VCFD+**	**VCFD+**	**VCFD+**
**Regarding VCI and VCFD**	**T0<br>baseline**	**T1<br>1-year**	**T2<br>2-years**
12--Vertebral corner inflammation (VCI) but not vertebral corner fat (VCFD) at any timepoint	**VCI+**<br>**VCFD-**	VCI+/-<br>**VCFD-**	VCI+/-<br>**VCFD-**
	VCI+/-<br>**VCFD-**	**VCI+**<br>**VCFD-**	VCI+/-<br>**VCFD-**
	VCI+/-<br>**VCFD-**	VCI+/-<br>**VCFD-**	**VCI+**<br>**VCFD-**
13--VCFD but not VCI at any timepoint	**VCFD+**<br>**VCI-**	VCFD+/-<br>**VCI-**	VCFD+/-<br>**VCI-**
	VCFD+/-<br>**VCI-**	**VCFD+**<br>**VCI-**	VCFD+/-<br>**VCI-**
	VCFD+/-<br>**VCI-**	VCFD+/-<br>**VCI-**	**VCFD+**<br>**VCI-**
14--VCI preceded VCFD	**VCI+**<br>**VCFD-**	**VCI-**<br>**VCFD+**	**VCI-**<br>VCFD+/-
	**VCI+**<br>**VCFD-**	**VCI-**<br>VCFD+/-	**VCI-**<br>**VCFD+**
	VCI+/-<br>**VCFD-**	**VCI+**<br>**VCFD-**	**VCI-**<br>**VCFD+**
15--VCI preceded or coincides with VCFD. VCFD does not precede VCI	**VCI+**<br>**VCFD+**	**VCI-**<br>VCFD+/-	**VCI-**<br>VCFD+/-
	**VCI+**<br>VCFD+/-	VCI+/-<br>**VCFD+**	**VCI-**<br>VCFD+/-

	**VCI+**<br>VCFD+/-	VCI+/-<br>VCFD+/-	VCI+/-<br>**VCFD+**
	VCI+/-<br>**VCFD-**	**VCI+**<br>**VCFD+**	**VCI-**<br>VCFD+/-
	VCI+/-<br>**VCFD-**	**VCI+**<br>VCFD+/-	VCI+/-<br>**VCFD+**
	VCI+/-<br>**VCFD-**	VCI+/-<br>**VCFD-**	**VCI+**<br>**VCFD+**
16--Sequential or simultaneous presence of VCI and VCFD across the three timepoints	**VCI+**<br>**VCFD+**	VCI+/-<br>VCFD+/-	VCI+/-<br>VCFD+/-
	VCI+/-<br>VCFD+/-	**VCI+**<br>**VCFD+**	VCI+/-<br>VCFD+/-
	VCI+/-<br>VCFD+/-	VCI+/-<br>VCFD+/-	**VCI+**<br>**VCFD+**
	**VCI+**<br>VCFD+/-	VCI+/-<br>**VCFD+**	VCI-<br>VCFD+/-
	**VCI+**<br>VCFD+/-	VCI+/-<br>VCFD+/-	VCI+/-<br>**VCFD+**
	VCI+/-<br>**VCFD+**	VCI+/-<br>VCFD+/-	**VCI+**<br>VCFD+/-
	VCI+/-<br>VCFD+/-	VCI+/-<br>**VCFD+**	**VCI+**<br>VCFD+/-
17—Coexistence of VCI and VCFD at the same timepoint	**VCI+**<br>**VCFD+**	**VCI-**<br>**VCFD-**	**VCI-**<br>**VCFD-**
	**VCI-**<br>**VCFD-**	**VCI+**<br>**VCFD+**	**VCI-**<br>**VCFD-**
	**VCI-**<br>**VCFD-**	**VCI-**<br>**VCFD-**	**VCI+**<br>**VCFD+**
	**VCI-**<br>**VCFD-**	**VCI+**<br>**VCFD+**	**VCI+**<br>**VCFD+**
	**VCI+**<br>**VCFD+**	**VCI+**<br>**VCFD+**	**VCI+**<br>**VCFD+**
	**VCI+**<br>**VCFD+**	**VCI+**<br>**VCFD+**	**VCI-**<br>**VCFD-**
	**VCI+**<br>**VCFD+**	**VCI-**<br>**VCFD-**	**VCI+**<br>**VCFD+**
18—Absence of VCI and VCFD	**VCI-**<br>**VCFD-**	**VCI-**<br>**VCFD-**	**VCI-**<br>**VCFD-**

The table presents definitions of patterns of MRI lesions over time on the vertebral corner level. The variables indicating presence of a pattern are coded as true (1) or false (0). MRI lesions are vertebral corner inflammation (VCI) and vertebral corner fat deposition (VCFD) and can be present (+), absent (-) or allowed to be either present or absent (+/-). The pattern variable is true for an MRI reader if the pattern of MRI lesions on a vertebral corner scored by that reader matches with at least one row of possible MRI patterns. When a pattern is scored by ≥2 MRI readers the pattern is deemed present and coded as true (1).

Supplementary table 2: baseline demographics

	**N=50**
Age at inclusion (mean (SD))	49 (9.8)
Sex (males)	43 (86%)
Uveitis	14 (28%)
Psoriasis	3 (6%)
IBD	3 (6%)
Heel enthesitis	7 (14%)
Dactylitis	3 (6%)
Peripheral arthritis	7 (14%)
HLA-B27 status	39 (78%)
Elevated CRP or ESR	27 (54%)
Number of syndesmophytes on radiographs reader 1 (mean (SD))	5.7 (4.5)
Number of syndesmophytes on radiographs reader 2 (mean (SD))	6.2 (5.1)
BASDAI (mean (SD))	3.8 (2.2)
ASDAS-CRP (mean (SD))	2.6 (1.2)
Physician global (mean (SD))	4.1 (2.1)
NSAID use current	33 (66%)
csDMARD use ever	12 (24%)
csDMARD use current	5 (10%)
bDMARD use ever	14 (28%)
bDMARD use current	11 (22%)

SpA features (uveitis, psoriasis, inflammatory bowel disease (IBD), heel enthesitis, dactylitis, peripheral arthritis, HLA-B27 and elevated CRP or ESR) are presented as ever/never. Numbers are presented as N (%) unless otherwise specified. SD, standard deviation; IBD, inflammatory bowel disease; CRP, C-reactive protein; ESR, erythrocyte sedimentation rate; BASDAI, Bath Ankylosing Spondylitis Disease Activity Index; ASDAS-CRP, Ankylosing Spondylitis Disease Activity Score; NSAID, non-steroidal anti-inflammatory drug; csDMARD, conventional synthetic Disease Modifying Anti Rheumatic Drug; bDMARD, biological Disease Modifying Anti Rheumatic Drug.
